# Endothelium-specific deletion of amyloid-β precursor protein exacerbates endothelial dysfunction induced by aging

**DOI:** 10.18632/aging.203401

**Published:** 2021-08-12

**Authors:** Livius V. d’Uscio, Zvonimir S. Katusic

**Affiliations:** 1 Departments of Anesthesiology and Perioperative Medicine, Mayo Clinic, Rochester, MN 55902, USA

**Keywords:** amyloid precursor protein, endothelial nitric oxide synthase, aging, endothelium, prostaglandins

## Abstract

The physiological function of amyloid precursor protein (APP) in the control of endothelial function during aging is unclear. Aortas of young (4-6 months old) and aged (23-26 months old) wild-type (WT) and endothelium-specific APP-deficient (eAPP^−/−^) mice were used to study aging-induced changes in vascular phenotype. Unexpectedly, aging significantly increased protein expression of APP in aortas of WT mice but not in aortas of eAPP^−/−^ mice thereby demonstrating selective upregulation APP expression in vascular endothelium of aged aortas. Most notably, endothelial dysfunction (impairment of endothelium-dependent relaxations) induced by aging was significantly exacerbated in aged eAPP^−/−^ mice aortas as compared to age-matched WT mice. Consistent with this observations, endothelial nitric oxide synthase (eNOS) protein expression was significantly decreased in aged eAPP^−/−^ mice as compared to age matched WT mice. In addition, protein expression of cyclooxygenase 2 and release of prostaglandins were significantly increased in both aged WT and eAPP^−/−^ mice. Notably, treatment with cyclooxygenase inhibitor, indomethacin, normalized endothelium-dependent relaxations in aged WT mice, but not in aged eAPP^−/−^ mice. In aggregate, our findings support the concept that aging-induced upregulation of APP in vascular endothelium is an adaptive response designed to protect and preserve expression and function of *eNOS*.

## INTRODUCTION

Vascular endothelium releases several factors involved in the local regulation of vascular tone and modulation of pro-inflammatory molecule production [1]. Among them are endothelium-derived relaxing factors in particular nitric oxide (NO) as well as endothelium-derived contracting factors such as prostaglandins and superoxide anion [2, 3]. Constitutive expression of endothelial nitric oxide synthase (eNOS) is mainly responsible for NO production in endothelial cells of conduit arteries [4, 5]. On the other hand, cyclooxygenase (COX) is a rate-limiting enzyme in the biosynthesis of prostaglandins. COX1 is constitutively expressed and plays an important role in vascular homeostasis, while COX2 is considered an inducible enzyme in mouse conduit arteries [6].

Aging of blood vessels are major contributors to the development of cardiovascular disease [7]. Existing evidence obtained in human and animal studies suggest that the earliest detectable vascular phenotype in aging involves the development of endothelial dysfunction, which is commonly characterized by the reduced endothelial production of NO and by the increased production of COX-derived vasoconstrictor factors [8–10]. Moreover, clinical studies have reported that endothelium-dependent vasodilatation progressively declines with age and occurs earlier in men than in women [11, 12].

Amyloid-β precursor protein (APP) is an evolutionarily conserved protein and is implicated in the development of Alzheimer's disease [13–15]. However, the exact function of APP outside the central nervous system, particularly in peripheral tissues, remains poorly understood [16–18]. We and others have shown that APP is expressed in cultured endothelial cells as well as in vascular wall of wild-type mice [19–24]. Under physiological conditions, APP is constitutively cleaved at the cell surface by α-secretase via non-amyloidogenic processing pathway. Alpha-processing of APP causes release of soluble APP alpha (sAPPα) ectodomain in the lumen of blood vessel wall [25–30]. Recently, we showed that endothelium-specific inactivation of APP (eAPP^−/−^) caused endothelial dysfunction in mice cerebral arteries [29]. However, no previous studies tested the effects of aging on vascular APP expression in systemic large conduit arteries. In the present study, we advance the hypothesis that APP exerts vascular protective effects on endothelial function during aging.

## RESULTS

### Characterization of mice

Body weight was increased in both aged wild-type (WT) and eAPP^−/−^ mice (P<0.05 vs. respective young mice; Table 1). Blood pressure measurements revealed that systolic blood pressure (SBP), mean blood pressure (MBP), and diastolic blood pressure (DBP) were unchanged in young and aged WT and eAPP^−/−^ mice (P>0.05; Table 1). Blood glucose was also not different between eAPP^−/−^ mice and their WT irrespective of age (Table 1). Lipid profile was unchanged in plasma of young WT and eAPP^−/−^ mice (P>0.05; Table 1). Aging caused a slight but significant increase in circulating levels of cholesterol in WT mice (P<0.05 vs. young WT; Table 1). In contrast, levels of triglycerides were decreased in aged eAPP^−/−^ mice (P<0.05 vs. young eAPP^−/−^ mice; Table 1). Plasma levels of norepinephrine were significantly increased in aged WT and eAPP^−/−^ mice (P<0.05 vs. respective young mice; Table 1). Aging significantly increased plasma levels of sAPPα in both WT and eAPP^−/−^ mice however, levels of sAPPα remained significantly decreased in eAPP^−/−^ mice as compared to age-matched WT mice (P<0.05; Table 1). However, plasma levels of amyloid-β 1-40 (Aβ_1-40_) were unaltered in young and aged WT and eAPP^−/−^ mice (P>0.05; Table 1).

**Table 1:** Characteristics of young and aged eAPP^−/^^−^ mice.

**Parameters**	**Young**			**Aged**	
	**WT littermates**	**eAPP^−/−^**		**WT littermates**	**eAPP^−/−^**
Body weight (g)	30±1 (10)	29±1 (10)		32±1 (12) *	33±1 (12) *
SBP (mmHg)	121±2 (9)	119±2 (9)		120±2 (9)	121±2 (11)
MBP (mmHg)	93±1 (9)	92±1 (9)		94±2 (9)	95±2 (11)
DBP (mmHg)	79±1 (9)	78±1 (9)		81±2 (9)	82±2 (11)
Glucose (mg/dL)	147±8 (10)	156±11 (10)		133±7 (12)	155±10 (12)
Cholesterol (mg/dL)	63±2 (11)	63±3 (11)		88±14 (11) *	72±3 (11)
HDL (mg/dL)	54± 2 (11)	52± 3 (11)		68±13 (11)	54±4 (11)
Triglycerides (mg/dL)	85± 5 (11)	91± 6 (11)		74±8 (11)	63±5 (11) *
Norepinephrine (pg/mL)	1599±222 (16)	1898±280 (16)		3601±1019 (9) *	3371±399 (7) *
sAPPα (pg/mL)	549±39 (10)	244±27 (10) †		734±72 (12) *	428±55 (12) *†
Aβ_1-40_ (pg/mL)	90±8 (10)	88±10 (10)		93±11 (8)	92±20 (8)

SBP indicates systolic blood pressure; MBP, mean blood pressure; DBP, diastolic blood pressure; HDL, high-density lipoprotein; WT, wild-type. Data are means ± SEM and the numbers of mice are indicated in the parentheses. * P<0.05 vs. young mice of same strain; † P<0.05 vs. age-matched WT littermates (two-way ANOVA followed by Tukey’s HSD test).

### APP expression and processing in aorta

**Figure 1:**
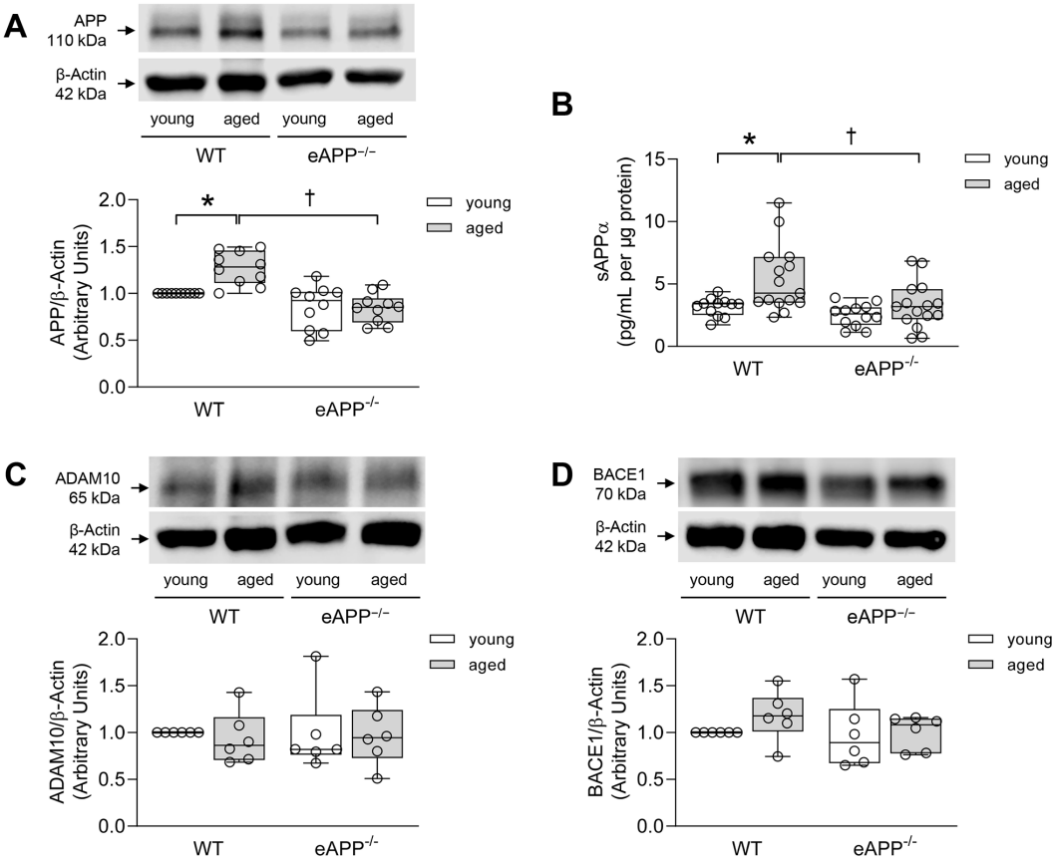
(**A**) Effects of aging on protein expression of APP in the aortas of wild-type (WT) littermates and eAPP^−/−^ mice (n=10 per group). (**B**) Effects of aging on *ex-vivo* sAPPα secretion from wild-type (WT) littermates and eAPP^−/−^ mice aortas. The supernatants were collected and analyzed for sAPPα levels. Results were normalized against tissue protein levels (n=12 per group for young WT littermates and eAPP^−/−^ mice and n=15 per group for aged WT littermates and eAPP^−/−^ mice). (**C**) Effects of aging on protein expression of ADAM10 in the aortas of wild-type (WT) littermates and eAPP^−/−^ mice (n=6 per group). (**D**) Effects of aging on protein expression of BACE1 in the aortas of wild-type (WT) littermates and eAPP^−/−^ mice (n=6 per group). Western blot results are the relative densitometry compared with β-actin protein. All results are representing box plots with whiskers showing the median, 25^th^ to 75^th^ percentiles, and min-max range. * P<0.05 versus young mice of same strain; † P<0.05 versus age-matched WT littermates (two-way ANOVA followed by Tukey’s HSD test).

Western blot analyses revealed that APP expression was significantly lower (by 27%) in the aorta of young eAPP^−/−^ mice (P<0.05 vs. young WT; Supplementary Figure 1). Aging caused increase in APP expression in WT mice aortas (P<0.05 vs. young WT; Figure 1A) while APP expression was unchanged in aged eAPP^−/−^ mice aortas (P>0.05 vs. young eAPP^−/−^ mice; Figure 1A). Release of sAPPα from the aorta was significantly increased in aged WT mice (P<0.05 vs. young WT mice; Figure 1B). In contrast, sAPPα release was not significantly affected by aging of eAPP^−/−^ mice (P>0.05 vs. young eAPP^−/−^ mice; Figure 1B). Release of Aβ_1-40_ from the aorta was not altered in young and aged WT and eAPP^−/−^ mice (P>0.05; Supplementary Figure 2). Moreover, protein expressions of a disintegrin and metalloproteinase domain-containing protein 10 (ADAM10) and beta-site APP cleaving enzyme 1 (BACE1) were unaltered in WT and eAPP^−/−^ mice irrespective of age (P>0.05; Figures 1C, 1D, respectively).

### Vascular contraction responses

Contractions to KCl were significantly increased in aged WT and eAPP^−/−^ mice aortas as compared to young mice (P<0.05; Table 2) while there were no differences between age-matched WT and eAPP^−/−^ mice (P>0.05; Table 2).

**Table 2:** Efficacy and potency of vasoconstrictors in young and aged eAPP^−/^^−^ mice aortas.

**Parameters**	**Young**			**Aged**	
	**WT littermates**	**eAPP^−/−^**		**WT littermates**	**eAPP^−/−^**
**KCl (g):**	1.95±0.06 (18)	1.87±0.04 (18)		2.53±0.11 (16) *	2.38±0.08 (16) *
**PGF_2α_:**					
E_max_ (%)	139±4 (9)	137±3 (9)		123±3 (8) *	124±4 (8) *
pEC_50_ (−log mol/L)	5.49±0.03 (9)	5.34±0.04 (9) †		5.69±0.03 (8) *	5.57±0.04 (8) * †
**Phenylephrine:**					
E_max_ (%)	63±6 (9)	50±5 (9)		41±8 (8) *	33±9 (8)
pEC_50_ (−log mol/L)	7.03±0.02 (9)	6.98±0.02 (9)		6.74±0.07 (8) *	6.68±0.05 (8) *

E_max_ indicates maximal efficacy; pEC_50_, potency; WT, wild-type. Data are means ± SEM and the numbers of mice are indicated in the parentheses. * P<0.05 vs. young mice of same strain; † P<0.05 vs. WT littermates of same age (two-way ANOVA followed by Tukey’s HSD test).

**Figure 2:**
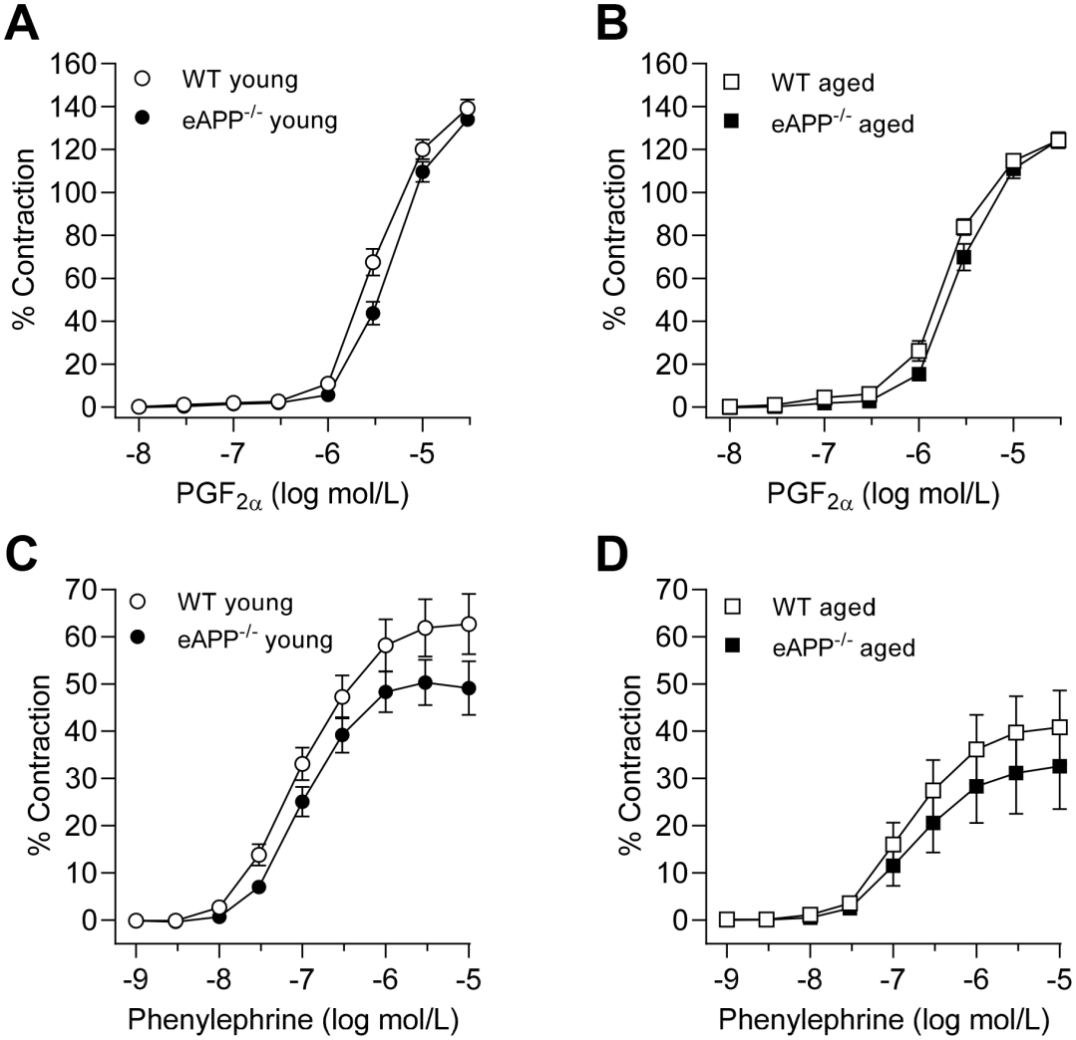
Concentration-dependent contractions to PGF_2α_ (**A**, **B**) and phenylephrine (**C**, **D**) in isolated aortic rings derived from young and aged eAPP^−/−^ mice and their respective wild-type (WT) littermates. Results are shown as mean ± SEM (n=9 per group for young WT littermates and eAPP^−/−^ mice and n=8 per group for aged WT littermates and eAPP^−/−^ mice) and contractions are expressed as percentage of response to a second KCl (80 mmol/L). No significant differences were detected between WT littermates and eAPP^−/−^ mice at same age (ANOVA with Bonferroni's correction).

Sensitivity to prostaglandin F_2α_ (PGF_2α_)-induced contractions was slightly lower in aortas of young and aged eAPP^−/−^ mice (P<0.05 as compared to their respective WT mice; Figures 2A, 2B, respectively; Table 2). In contrast, PGF_2α_ potency was significantly increased in aged WT and eAPP^−/−^ mice (P<0.05 vs. respective young mice; Table 2), and efficacy of PGF_2α_ was significantly decreased in aged WT and eAPP^−/−^ mice (P<0.05 vs. respective young mice; Table 2).

While no significant differences between age-matched WT and eAPP^−/−^ mice were observed, the potency of phenylephrine was reduced in aged WT and eAPP^−/−^ mice (P<0.05 vs. respective young mice; Table 2; Figure 2C, 2D). Furthermore, the efficacy was significantly reduced in aged WT mice (P<0.05 vs. young WT mice; Table 2) while it tended to be reduced in aged eAPP^−/−^ mice (P=0.088; Table 2).

### Vascular endothelial function

**Figure 3:**
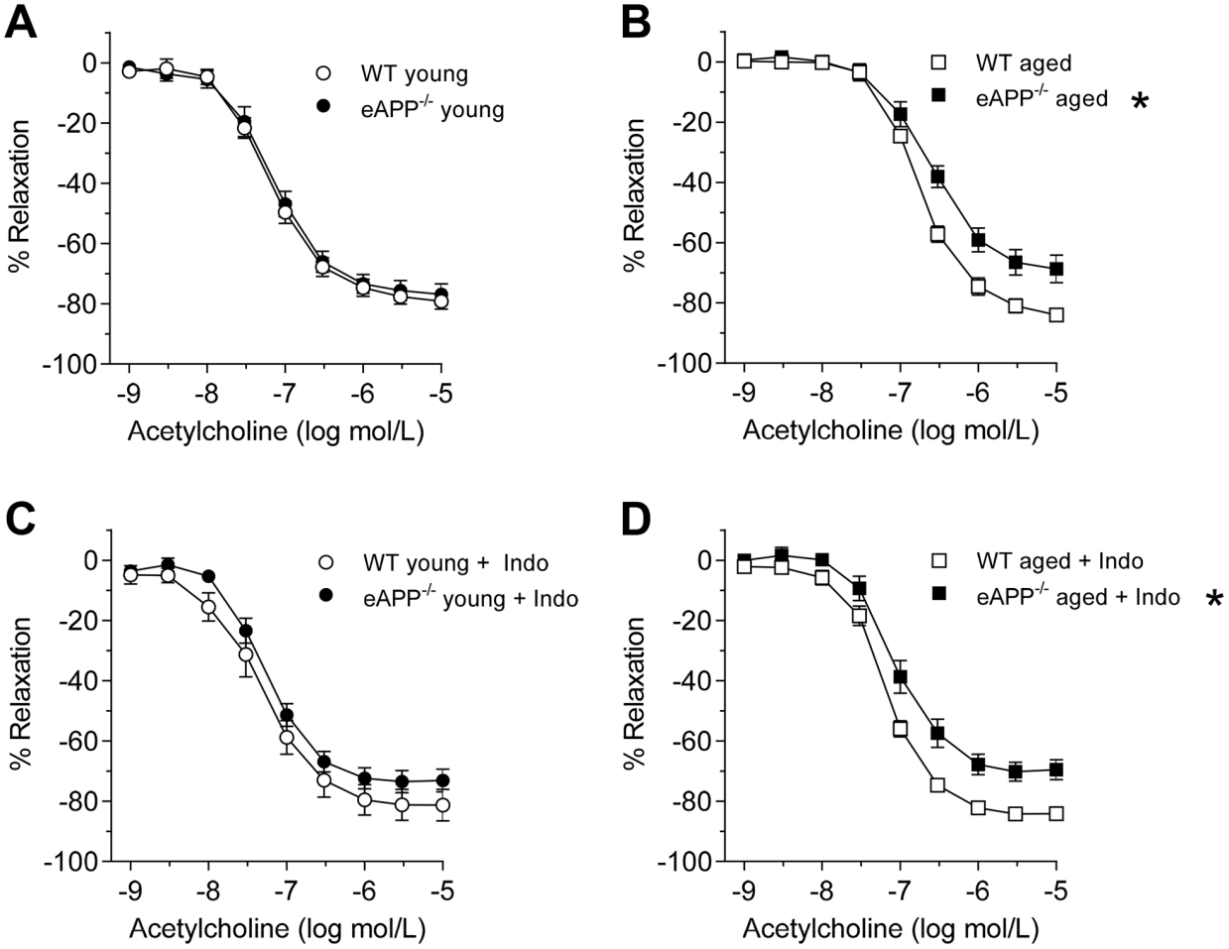
Endothelium-dependent relaxations to ACh in isolated aorta from young (**A**; n=9 per group) and aged (**B**; n=8 per group) wild-type (WT) littermates and eAPP^−/−^ mice aortas. Effects of indomethacin (Indo; 10^-5^ mol/L) on responses to ACh in young (**C**; n=9 per group) and aged (**D**; n=7 per group) WT littermates and eAPP^−/−^ mice aortas. Results are shown as mean ± SEM and expressed as percent relaxation from submaximal contractions induced by PGF_2α_. * P>0.05 versus wild-type littermates (ANOVA with Bonferroni's correction).

Acetylcholine (ACh)-induced endothelium-dependent relaxations were unaltered in young mice with endothelial-specific APP deletion (P>0.05 vs. young WT littermates; Figure 3A and Table 3). Aging caused a significant impairment to ACh in WT mice (P<0.05 vs. young WT mice; Table 3) while the efficacy was unaltered (Table 3). Furthermore, relaxations to ACh were further decreased in aged eAPP^−/−^ mice (P<0.05 vs. aged WT mice; Figure 3B and Table 3).

**Table 3:** Efficacy and potency of endothelium-dependent relaxations in young and aged eAPP^−/^^−^ mice aortas.

**Parameters**	**Young**			**Aged**	
	**WT littermates**	**eAPP^−/−^**		**WT littermates**	**eAPP^−/−^**
**E_max_ (%):**					
ACh	78±2 (9)	76±4 (9)		83±2 (8)	69±5 (8) †
ACh + Indomethacin	81±6 (9)	73±4 (9)		84±2 (7)	70±3 (7) †
**pEC_50_ (−log mol/L):**					
ACh	7.18±0.04 (9)	7.16±0.05 (9)		6.75±0.01 (8) *	6.61±0.05 (8) * †
ACh + Indomethacin	7.33±0.08 (9)	7.25±0.04 (9)		7.17±0.03 (7) #	7.04±0.06 (7) * #

E_max_ indicates maximal efficacy; pEC_50_, potency; WT, wild-type. Data are means ± SEM and the numbers of mice are indicated in the parentheses. * P<0.05 vs. young mice of same strain; † P<0.05 vs. WT littermates of same age (two-way ANOVA followed by Tukey’s HSD test); # P<0.05 vs. without indomethacin treatment (unpaired t-test).

Incubation of aortas with indomethacin did not affect relaxations to ACh in young mice (P>0.05; Figure 3C). In contrast, indomethacin significantly improved endothelium-dependent relaxations to ACh in both aged WT and eAPP^−/−^ mice (P<0.05 vs. aortas without indomethacin; Table 3). However, the efficacy is still significantly reduced in aged eAPP^−/−^ mice in the presence of indomethacin (P<0.05 vs. aged WT mice with indomethacin; Figure 3D and Table 3). Blockade of NOS with *N^ω^*-nitro L-arginine methyl ester (L-NAME) in the presence of indomethacin completely blocks relaxations to ACh in both young and aged WT and eAPP^−/−^ aortas (Supplementary Figure 3).

### *Ex-vivo* production of prostaglandins in aorta

**Figure 4:**
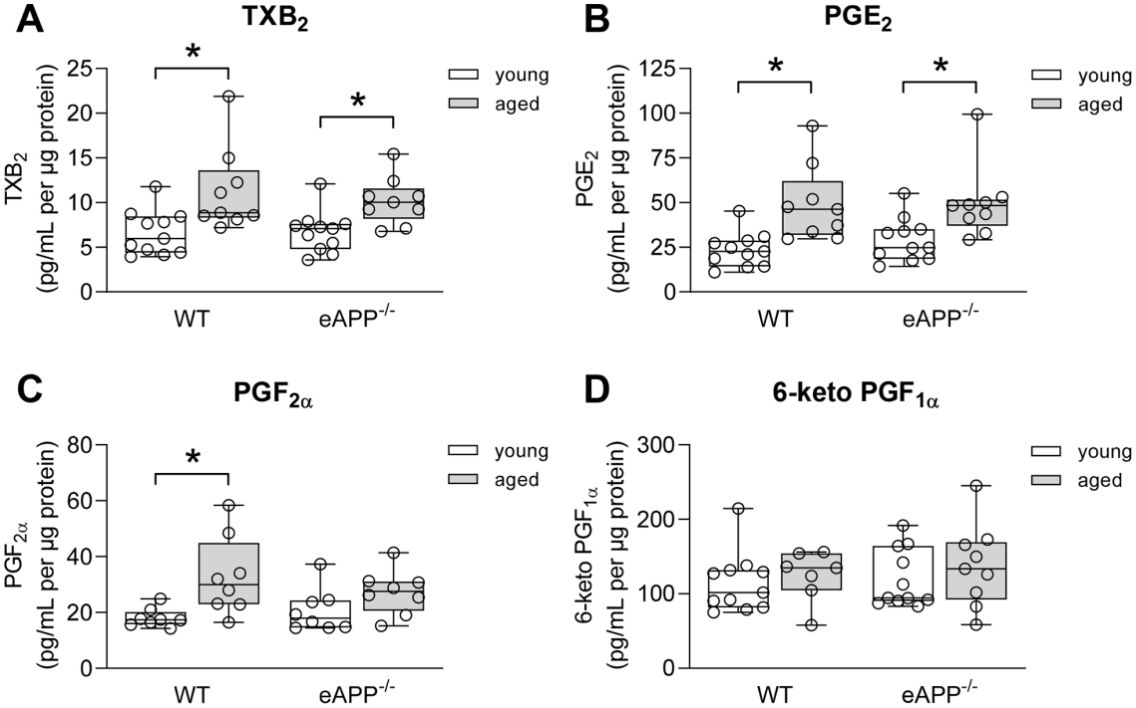
**Effects of aging on *ex-vivo* secretion of prostaglandins from wild-type (WT) littermates and eAPP^−/−^ mice aortas.** The supernatants were collected and analyzed for TXB_2_ (**A**; n=11 per group for young mice and n=9 per group for aged mice), PGE_2_ (**B**; n=11 per group for young mice and n=9 per group for aged mice); PGF_2α_ (**C**; n=8 per group for young mice and n=8 per group for aged mice); and 6-keto PGF_1α_ (**D**; n=11 per group for young mice and n=7-9 per group for aged mice). All results were normalized against tissue protein levels and are representing box plots with whiskers showing the median, 25^th^ to 75^th^ percentiles, and min-max range. * P<0.05 versus young mice of same strain (two-way ANOVA followed by Tukey’s HSD test).

We next determined whether age-related changes in vascular prostaglandins contribute to the observed decline in endothelial function of aortas, we measured release of prostaglandins in conditioned medium. Thromboxane B_2_ (TXB_2_) and prostaglandin E_2_ (PGE_2_) productions were significantly increased in both aged WT and eAPP^−/−^ mice (P<0.05 vs. young mice; Figure 4A, 4B) while production of PGF_2α_ was significantly increased only in aged WT mice (P<0.05 vs. young WT; Figure 4C). In contrast, production of 6-keto PGF_1α_ (a stable metabolite of prostaglandin I_2_) was not different among young and aged WT and eAPP^−/−^ mice (P>0.05; Figure 4D).

**Figure 5:**
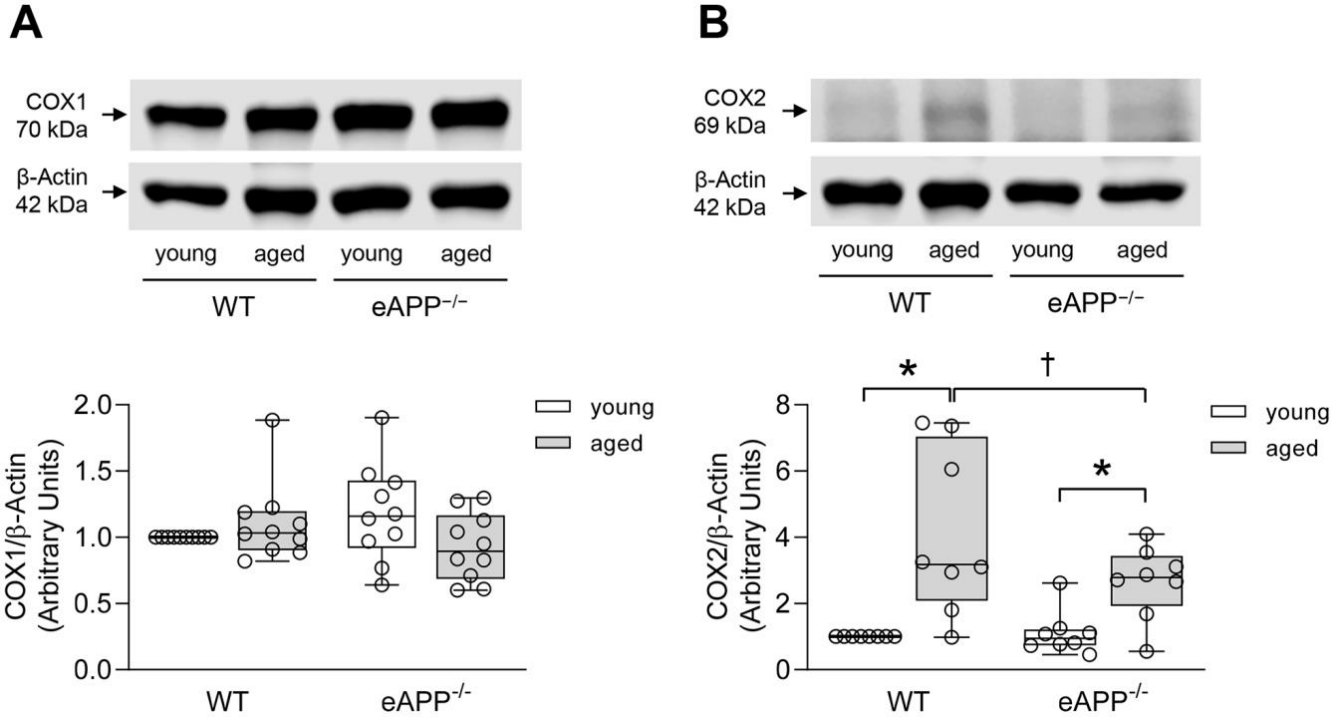
Protein expressions of COX1 (**A**) and COX2 (**B**) in the aortas of wild-type (WT) littermates and eAPP^−/−^ mice. Western blot results are the relative densitometry compared with β-actin protein (n=10 per group for COX1 and n=8 per group for COX2). All results are representing box plots with whiskers showing the median, 25^th^ to 75^th^ percentiles, and min-max range. * P<0.05 versus young mice of same strain; † P<0.05 versus age-matched WT littermates (two-way ANOVA followed by Tukey’s HSD test).

We also performed western blot analyses for protein expression of COX isoforms in aortas. COX1 expression was not different between WT and eAPP^−/−^ mice irrespective of age (P>0.05; Figure 5A). In contrast, protein expression of COX2 was significantly increased in both aged WT and eAPP^−/−^ mice (P<0.05 vs. young mice; Figure 5B). Moreover, the increase in COX2 was significantly reduced in aged eAPP^−/−^ mice (P<0.05 vs. aged WT mice; Figure 5B).

### Protein expression of eNOS

**Figure 6:**
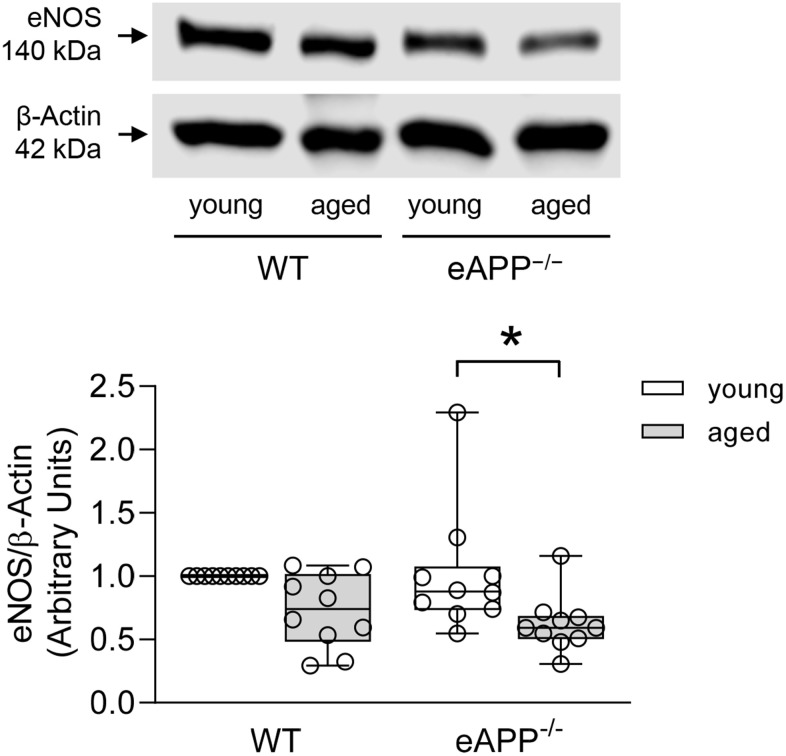
**Effects of aging on protein expression of eNOS in the aortas of wild-type (WT) littermates and eAPP^−/−^ mice.** Western blot results are the relative densitometry compared with β-actin protein (n=10 per group). All results are representing box plots with whiskers showing the median, 25^th^ to 75^th^ percentiles, and min-max range. * P<0.05 versus young mice of same strain (two-way ANOVA followed by Tukey’s HSD test).

Expression of eNOS was significantly decreased in the aorta of aged eAPP^−/−^ mice (P<0.05 vs. young eAPP^−/−^ mice; Figure 6). Furthermore, eNOS protein tended to be reduced in aged WT mice but it did not reach statistical significance (Figure 6).

### Superoxide anion

**Figure 7:**
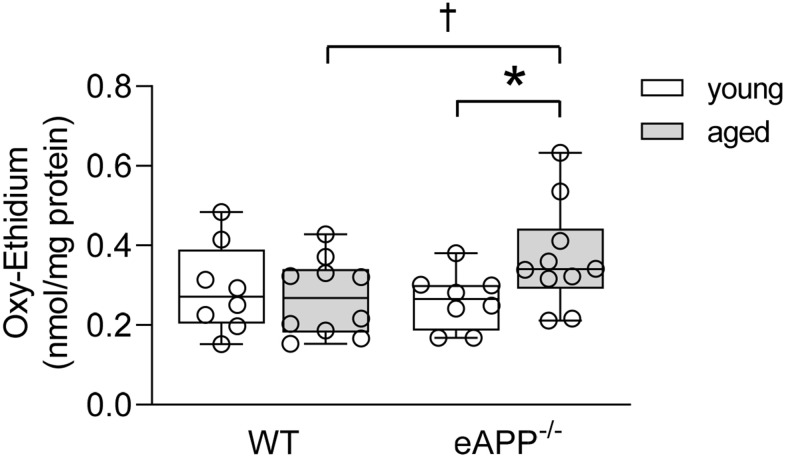
**Quantitative HPLC analysis of superoxide anion in aortas from young and aged wild-type (WT) littermates and eAPP^−/−^ mice aortas.** All results were normalized against tissue protein levels and are representing box plots with whiskers showing the median, 25^th^ to 75^th^ percentiles, and min-max range (n=8 per group for young WT littermates and eAPP^−/−^ mice and n=10 per group for aged WT littermates and eAPP^−/−^ mice). * P<0.05 versus young mice of same strain; † P<0.05 versus age-matched WT littermates (two-way ANOVA followed by Tukey’s HSD test).

To investigate whether endothelial dysfunction in aging was caused by increased superoxide anion production in aorta, we performed HPLC-based assay of 2-hydroxyethidium. Levels of superoxide anion were significantly enhanced in aged eAPP^−/−^ mice (P<0.05 vs. young eAPP^−/−^ mice; Figure 7) while superoxide anion levels were unaltered in aged WT mice (P>0.05 vs. young WT mice; Figure 7).

### Intracellular cGMP levels

**Figure 8:**
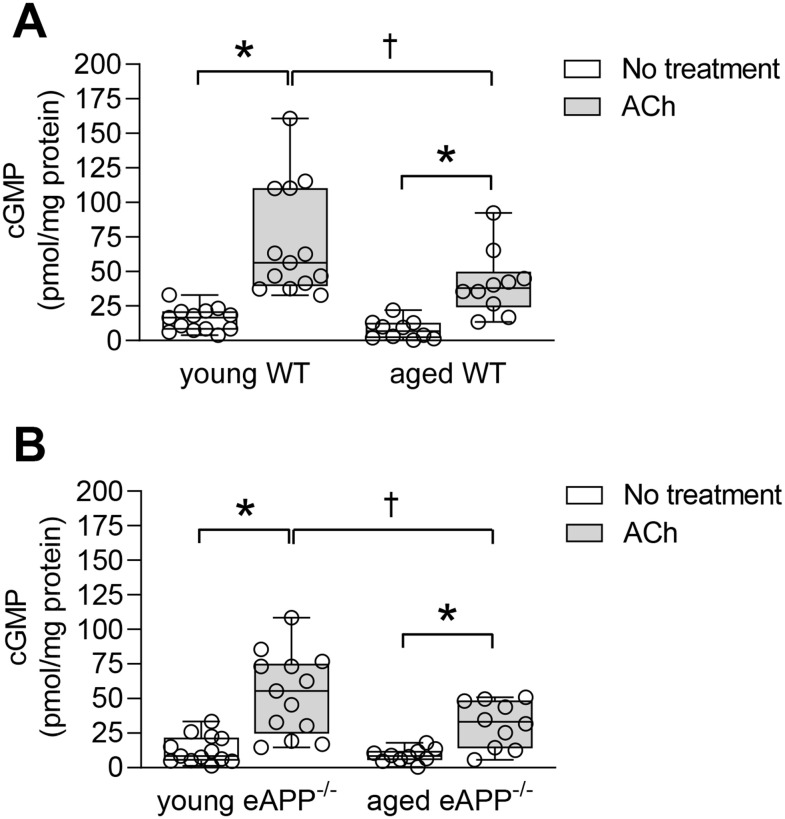
Quantitative analysis of cGMP in aortas from young and aged wild-type (WT) littermates aortas (**A**) and eAPP^−/−^ mice aortas (**B**) under basal and ACh (10 μM) stimulated conditions. All results were normalized against tissue protein levels and are representing box plots with whiskers showing the median, 25^th^ to 75^th^ percentiles, and min-max range (n=13 per group for young WT littermates and eAPP^−/−^ mice and n=10 per group for aged WT littermates and eAPP^−/−^ mice). * P<0.05 versus basal levels; † P<0.05 versus young WT mice (two-way ANOVA followed by Tukey’s HSD test).

Under basal conditions, cyclic guanosine 3',5'-monophosphate (cGMP) were unchanged in aortas of WT and eAPP^−/−^ mice irrespective of age (P>0.05; Figure 8). ACh stimulated cGMP levels were significantly increased in young and aged WT and eAPP^−/−^ mice (P<0.05 vs. basal levels; Figure 8). However, ACh stimulated cGMP levels were significantly decreased in aged WT and eAPP^−/−^ mice (P<0.05 young mice in the presence of ACh; Figure 8). In contrast, cGMP levels did not differ between WT and eAPP^−/−^ mice groups under basal and stimulated conditions (P>0.05; Figure 8).

### *Ex-vivo* studies with cytokines

**Figure 9:**
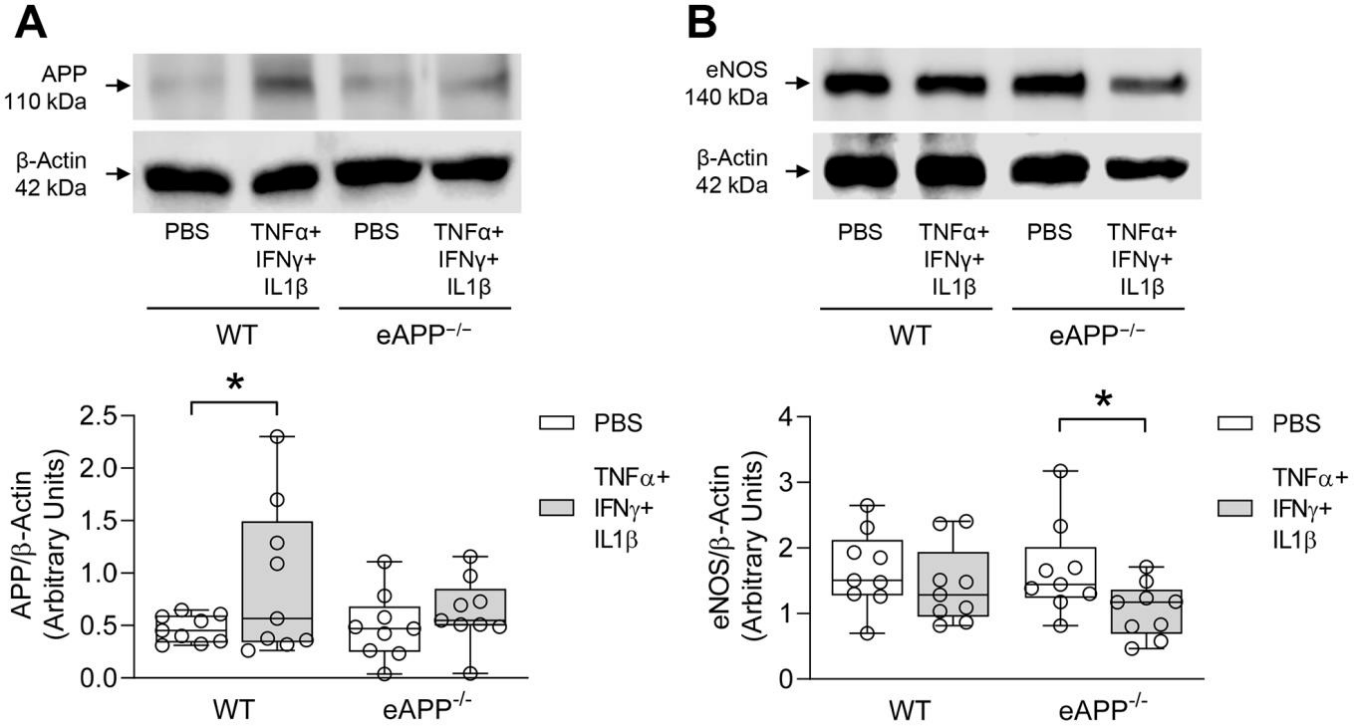
Effects of *ex-vivo* treatment for 24 hours with cytokines cocktail on protein expressions of APP (**A**) and eNOS (**B**) in the aortas of young wild-type (WT) littermates and eAPP^−/−^ mice. Western blot results are the relative densitometry compared with β-actin protein (n=9 per group). All results are representing box plots with whiskers showing the median, 25^th^ to 75^th^ percentiles, and min-max range. * P<0.05 versus control PBS treatment (two-way ANOVA followed by Tukey’s HSD test).

We next attempted in different experimental conditions to provide insight into the effects of APP deletion on eNOS expression in vascular endothelium. Young WT and eAPP^−/−^ mice aortas were used to examine the effects of pro-inflammatory cytokines cocktail on expression of APP and eNOS. Western blot analysis revealed that APP protein was significantly increased in young WT mice treated with cytokines (P<0.05 vs. PBS treatment; Figure 9A) while APP expression was not significantly affected in young eAPP^−/−^ mice (P>0.05; Figure 9A). In contrast, eNOS expression was significantly reduced in young eAPP^−/−^ but not in young WT aortas after cytokines treatment (P<0.05 vs. PBS treatment; Figure 9B). Expressions of iNOS and COX2 were equally increased in both groups of mice (P<0.05 vs. PBS treatment; Supplementary Figure 4).

## DISCUSSION

There are several major findings in this study. First, protein expression of endothelial APP and production of sAPPα in endothelium of aged WT mice are increased as compared to aged eAPP^−/−^ mice. Second, impairment of endothelium-dependent relaxations to acetylcholine induced by aging is exacerbated in eAPP^−/−^ mice aortas. Third, eNOS expression was decreased whereas levels of superoxide anion were enhanced in aortas of aged eAPP^−/−^ mice. Fourth, pro-inflammatory cytokines increased APP protein expression only in isolated aortas of young WT mice. In contrast, pro-inflammatory cytokines decreased expression of eNOS exclusively in young eAPP^−/−^ mice. Taken together, the results suggest that during aging endothelial APP exerts vascular protective effects. These effects appear to be mediated by preservation of eNOS and endothelium-dependent vasodilator function.

In the present study we demonstrated for the first time that APP expression and sAPPα release were increased in aged wild-type mice aortas. Importantly, these age-induced changes in expression and processing of APP were not observed in the aorta of eAPP^−/−^ mice thus demonstrating that endothelial cells are a major site of increased APP expression and production of sAPPα. Of note, Austin and colleagues reported that APP expression is increased mainly in intimal layer of both human and apolipoprotein E-deficient mice atherosclerotic aortas [21]. Importantly, protein expression of mature α-secretase ADAM10 was unchanged in aged aortas. Whether enhanced α-processing of APP could be explained by the increased enzymatic activity of ADAM10, or some other α-secretases, remains to be determined [27].

Prior study reported that hypertension causes shift towards amyloidogenic APP β-processing and development of cerebral amyloid angiopathy [31]. However, we detect neither blood pressure increases nor changes of aortic protein expression of β-secretase, BACE1, and production of Aβ_1-40_ in aged wild-type and eAPP^−/−^ mice indicating that amyloidogenic processing of APP in aorta is not affected by aging. Thus, our study demonstrates that upregulation of APP expression in aged wild-type mice is not induced by alterations of arterial blood pressure.

It is not clear what are the exact mechanisms responsible for increased expression and α-processing of APP in large conduit arteries of aged WT mice. Relevant to our observations, it is well known that aging is characterized by a state of chronic, low-grade inflammation [32, 33]. Moreover, expression of inflammatory cytokines such as TNFα, INFγ, and/or IL-1ß have been reported to be elevated in arteries of aged mice [34–37]. We speculate that the presence of proinflammatory stimuli is the most likely explanation for increased expression and non-amyloidogenic processing of APP. Consistent with this hypothesis, TNFα and IL-1ß increase APP expression and secretion of sAPPα from endothelial cells [19–21, 38]. In the present study, we also demonstrated that *ex-vivo* treatment of isolated aortas with cocktail of inflammatory cytokines led to increased APP expression in young WT mice but not in eAPP^−/−^ mice again indicating that endothelium is a major source of APP. The concentrations of cytokines used for *ex-vivo* studies were higher than published serum cytokines levels detected in-vivo in young control mice [39]. Whether aging significantly increases circulating levels of cytokines tested in our study remains to be determined. Nevertheless, it is conceivable that the observed upregulation of APP represents an adaptative/protective response of endothelial cells to aging-induced inflammation [40, 41].

Although plasma levels of sAPPα were lower in eAPP^−/−^ mice as compared to WT mice, aging increased sAPPα levels not only in WT mice but also in eAPP^−/−^ mice. This indicates that other cell types besides endothelial cells must contribute to elevation of circulating sAPPα. It is well known that APP is also expressed in platelets and cleaved by non-amyloidogenic APP processing thereby releasing sAPPα upon activation [38, 42, 43]. Notably, platelets count is significantly increased in aged wild-type C57BL/6J mice [44]. Increased release of sAPPα from platelets may help explain elevated circulating levels of sAPPα in aged eAPP^−/−^ mice.

Circulating levels norepinephrine were increased in both aged WT and eAPP^−/−^ mice despite normal blood pressure. This may explain the observed reduced responsiveness to phenylephrine in aging. Chronic exposure of the peripheral vasculature to high circulating levels of norepinephrine causes compensatory downregulation of reactivity to phenylephrine. Indeed, similar observations were made in healthy human subjects showing that α-adrenergic vasocontraction responsiveness to increased circulating norepinephrine levels is reduced with age in healthy men [45].

Aging also alters arachidonic acid metabolism in endothelial cells and these alterations can profoundly affect vasomotor function [46, 47]. Human and animal studies have established that enhanced production of vasoconstrictor prostaglandins can impair endothelium-dependent relaxations during aging [8, 10]. In subjects older than 60 years, the treatment with COX-inhibitor, indomethacin, potentiates endothelium-dependent vasodilation to ACh thus demonstrating that vasoconstrictor prostaglandins contribute to impairment of endothelium-dependent relaxations in humans [10]. In line with these studies, we presented evidence that indomethacin significantly improved endothelium-dependent relaxations in response to ACh in both aged WT and eAPP^−/−^ mice aortas. Consistent with these observations, production of TXA_2_ and PGE_2_ was significantly increased in both aged WT and eAPP^−/−^ mice. The reason why the production of PGF_2α_ was increased only in aged WT littermates is unclear and remains to be determined. However, this could be related to different expression levels of COX2 between aged WT and eAPP^−/−^ mice (see below). In contrast, the production of 6-keto PGF_1α_ remained unchanged in aged conduit arteries demonstrating that the metabolism of prostaglandin I_2_, which causes direct vasodilation in smooth muscle cells, is unaffected.

Interestingly, our study revealed that protein expression of COX2 was significantly increased in both aged wild-type and eAPP^−/−^ mice while protein expression of COX1 remained unchanged. Although COX1 primarily contributes to basal vascular production of prostaglandins, COX2 also contributes to the production of prostaglandins [48]. Indeed, it has been reported that COX2 is expressed in endothelial cells and that inhibition of eNOS unmasks the ability of ACh to elicit endothelium-dependent contractions which are sensitive to inhibition of COX2 [49]. Thus, increased production of vasoconstrictor prostaglandins by COX2 appears to be responsible for reduced endothelium-dependent relaxations in aged mice. Notably, this phenomenon was not affected by genetic deletion of APP in endothelium.

To determine the reason why endothelium-dependent relaxations to ACh were still impaired in aged eAPP^−/−^ mice despite treatment with indomethacin, we studied eNOS protein expression. Interestingly, eNOS protein was decreased in the aorta of aged eAPP^−/−^ mice but not in young eAPP^−/−^ mice. In contrast, expression of eNOS is unchanged in aged WT mice aortas and this is in line with the previous reports [50, 51]. Moreover, eNOS expression is not altered in conduit and resistance arteries of different mouse models of cardiovascular disease despite presence of inflammatory cytokines [52–54]. These observations are confirmed and further extended by our *ex-vivo* studies of isolated aortas treated with cytokines cocktail showing that eNOS expression was downregulated in young eAPP^−/−^ mice, but not in young WT mice. From these findings we concluded that during aging, the presence of APP in aortic vascular endothelium is essential for normal expression and function of eNOS.

**Figure 10:**
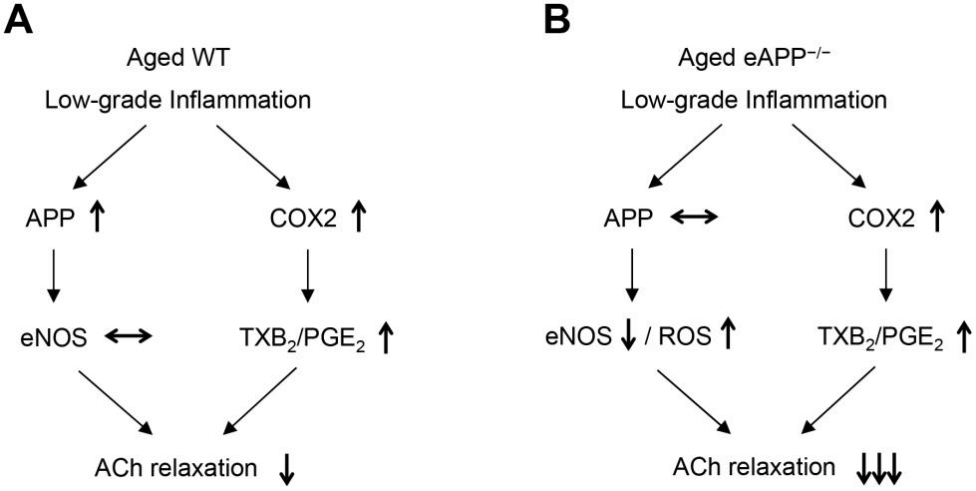
Schematic summary of the effects of aging on endothelial function in wild-type (WT) mice (**A**) and endothelium-specific amyloid precursor protein-deficient (eAPP^−/−^) mice (**B**). Please note that expression of APP is increased in aged wild-type mice (**A**) but not in aged eAPP^−/−^ mice (**B**) aortas. ↑ = increase; ↓ = decrease; ↔ = no change; eNOS = endothelial nitric oxide synthase; ROS = reactive oxygen species; COX2 = cyclooxygenase 2; TXB_2_ = thromboxane B_2_; PGE_2_ = prostaglandin E_2_; ACh = acetylcholine.

Superoxide anion levels were increased in aged eAPP^-/-^ aortas but not in aged WT aortas. Increased ROS generation and/or reduced antioxidant capacity could contribute to the increased levels of superoxide anion. However, insufficient NO production in aged eAPP^-/-^ mice can also lead to enhanced production of superoxide anion thereby further exacerbating impairment of endothelium-dependent relaxations (Figure 10). Indeed, several studies provided evidence that NO inactivated superoxide anion by rapid nonenzymatic reaction with superoxide anion [55–58].

We were unable to observe any changes in superoxide anion production in conduit arteries derived from aged WT mice under basal conditions and this is in line with existing literature [37, 51, 59]. In contrast, several studies reported increased levels of superoxide anion in aged wild-type mice arteries [50, 60, 61]. The reason for this discrepancy is unclear. One possible explanation is that vascular superoxide production is increased in very old (31 months old) wild-type mice [37, 61].

Elevation of aortic cGMP levels induced by ACh are decreased in both aged WT and eAPP^−/−^ mice as compared to young mice aortas while basal cGMP levels were not different between WT and eAPP^−/−^ mice. As mentioned earlier, the endothelium is a major source not only of NO but of prostaglandins as well [3]. Relevant to our observations, endogenous prostaglandins have been shown to decrease cGMP levels and to induce contractions in response to ACh in the aorta [62, 63] thus suggesting that exaggerated production of prostaglandins during aging can impair ACh-stimulated production of cGMP. At the present time we do not have an explanation as to why levels of cGMP were not decreased in aged eAPP^−/−^ mice.

In aggregate, the present study demonstrates that endothelial dysfunction (loss of NO) is more pronounced in aged eAPP^−/−^ mice than in aged WT littermates. We also provide evidence that the absence of APP in endothelial cells causes downregulation of eNOS expression in eAPP^−/−^ mice. Our findings support the concept that up-regulation of endothelial APP and production of sAPPα are adaptive mechanisms designed to protect blood vessel wall from detrimental effects of inflammation associated with aging. In this regard, preservation of NO production in endothelium of aged aortas appears to be critically important function of endothelial APP.

## MATERIALS AND METHODS

### Experimental animals

APP^flox/flox^;Tie2-Cre^−^ mice (wild-type [WT] littermates) and APP^flox/flox^;Tie2-Cre^+^ (eAPP^−/−^) mice were generated and genotyped in our laboratory as described previously [29]. All mice were maintained on 12h/12h light/dark cycle and on standard chow with free access to drinking water. Institutional Animal Care and Use Committee of the Mayo Clinic reviewed and approved the experimental design. In addition, the protocols complied with the guidelines of ARRIVE and NIH for care and use of laboratory animals. Young male mice (4-6 months old) and aged male mice (23-26 months old) were anaesthetized with overdose of pentobarbital (200-250 mg/kg BW, i.p.) and were exsanguinated using cardiac puncture method for collection of blood samples. The aortas were carefully harvested and were thereafter dissected free from surrounding connective tissues in cold (4° C) Krebs solution [composition (in mmol/L): NaCl 118.6; KCl 4.7; CaCl_2_ 2.5; MgSO_4_ 1.2; KH_2_PO_4_ 1.2; NaHCO_3_ 25.1; glucose 10.1; EDTA 0.026]. Age-matched WT littermate mice served as controls in studies designed to determine vascular phenotype characteristics of eAPP^−/−^ mice.

### Blood pressure

A tail-cuff technique (Harvard Apparatus Ltd., Kent, England) was used to monitor SBP and MBP [64]. DBP was calculated through the formula: DBP = [(3x*MBP*) - SBP]/2.

### Glucose and lipid levels

Blood was collected through cardiac puncture and was transferred to a tube containing EDTA. Glucose was determined immediately using Accu Check^®^ (Roche Diagnostics, Indianapolis, IN). Thereafter, blood samples were centrifuged for 10 min at 2000 rpm (4° C) and plasma was obtained and stored at -80° C. Plasma levels of cholesterol, HDL, and triglyceride were measured as described previously [24].

### Determination of plasma norepinephrine levels

Blood was placed in a tube containing EGTA with reduced glutathione. After centrifugation, plasma was stored at -80° C until assayed. After extraction with activated alumina norepinephrine levels were determined by HPLC with electrochemical detection as described [65].

### Determination of plasma sAPPα and Aβ_1-40_ levels

A highly sensitive sAPPα mouse/rat ELISA kit (Immuno-Biological Laboratories America, Minneapolis, MN) and Aβ_1-40_ mouse ELISA kit (Invitrogen, Camarillo, CA) were used to determine sAPPα and Aβ_1-40_ levels, respectively.

### Determination of *ex-vivo* release of sAPPα and Aβ_1-40_

Ten millimeters long aortas were opened longitudinally and were incubated in minimal essential medium (MEM) in the presence of bovine serum albumin (0.1%), penicillin (100 U/mL) and streptomycin (100 μg/mL; GIBCO^®^ Thermo Fischer Scientific Inc., Waltham, MA) for 24 hours at 37° C in CO_2_ incubator. Thereafter, the cultured medium was collected and stored at -80° C until assayed. Levels sAPPα and Aβ_1-40_ in conditioned medium were determined using the ELISA kits as described above and all results were normalized against protein levels of aortas.

### Organ chamber studies in isolated aortas

An isometric force measurement was used to study vasomotor function of isolated thoracic aortic rings as previously described [66]. Aortic rings were stretched to optimal force of 1.5 grams. Thereafter, the rings were contracted twice with KCl (80 mmol/L) and washed out. Concentration-dependent response curves to PGF_2α_ (10^-8^−3x10^-5^ mol/L; Cayman, Ann Arbor, MI) and L-phenylephrine (10^-9^−10^-5^ mol/L; Sigma, St. Louis, MO) were cumulatively obtained. After washing out, concentration-dependent responses to ACh (10^-9^−10^-5^ mol/L; Sigma) were recorded in parallel in the absence and in the presence of indomethacin (10^-5^ mol/L, 30 minutes; Sigma) and indomethacin + L-NAME (3x10^-4^ mol/L, 30 minutes; Sigma). Various concentrations of PGF_2α_ (3x10^-6^–8x10^-6^ mol/L) were used in order to achieve similar submaximal contractions in young WT and eAPP^−/−^ mice (57±5% and 61±5%, respectively; P>0.05; n=9) as well as in aged WT and eAPP^−/−^ mice (66±4% and 69±3%, respectively; P>0.05; n=8).

### Determination of *ex-vivo* release of prostaglandins

Ten millimeters long thoracic aortas were opened longitudinally and were incubated in modified MEM in the presence of 0.1% BSA, 100 U/mL penicillin and 100 μg/mL streptomycin for 24 hours (37° C in CO_2_ incubator). Thereafter, the cultured medium was collected and stored at -80° C. Commercially available colorimetric ELISA kits (Cayman, Ann Arbor, MI) were used for measurements of TXB_2_ (a stable metabolite of TXA_2_), PGE_2_, PGF_2α_, and 6-keto PGF_1α_ concentrations in the medium. All results were normalized against aortic protein levels.

### Determination of intracellular superoxide anions

Intracellular levels of superoxide anion were quantified in fresh aortic rings using 2-hydroxyethidium standard by HPLC-based fluorescence detection and normalized against tissue protein levels as described in detail in previous studies [67, 68].

### Determination of cGMP

Twelve millimeters long aortas were incubated for 2 hours at 37° C in MEM containing bovine serum albumin (0.1%), penicillin (100 U/mL) and streptomycin (100 μg/mL). A phosphodiesterase inhibitor 3-isobutyl-1-methylxanthine (100 μmol/L; Sigma) was also added to prevent degradation of cyclic nucleotides. At the end of incubation time, ACh (10 μM) was added for 2 minutes. Thereafter, all samples were immediately placed in liquid N_2_ and stored at -80° C until assayed. Colorimetric cGMP ELISA immunoassay (Cell Biolabs Inc., San Diego, CA) was used to determine cGMP levels and the results were normalized against aortic protein concentrations.

### *Ex-vivo* studies with cytokines

Ten millimeters long thoracic aortas were opened longitudinally and were incubated with control solution (PBS) or cytokines cocktail composing 20 ng/mL recombinant mouse tumor necrosis factor α (TNFα; R&D Systems, Minneapolis, MN), 50 ng/mL recombinant mouse interferon γ (INFγ; R&D Systems), and 1 ng/mL recombinant mouse interleukin-1ß (IL-1ß; R&D Systems) in MEM in the presence of bovine serum albumin (0.1%), penicillin (100 U/mL) and streptomycin (100 μg/mL) for 24 hours at 37° C in CO_2_ incubator [69]. Thereafter, the aortas were homogenized in lysis buffer for western blot analysis (see below).

### Western blot analysis

Dissected aortas were homogenized in lysis buffer using glass grinder and pestle 20 as previously described [68]. After centrifugation, protein levels were determined in supernatants using DC protein assay kit (BioRad, Hercules, CA). For each sample, 50 μg protein was loaded on 7.5% or 10% TGX SDS-PAGE gels (BioRad). Antibodies against APP (1:250; cat# 51-2700, Invitrogen, Carlsbad, CA), BACE1 (1:250; cat# S5606, Cell signaling, Danvers, MA), ADAM10 (1:500; cat# AB19026, Millipore, Burlington, MA), eNOS (1:250, cat# 610297, BD Biosciences, San Jose, CA), inducible nitric oxide synthase (iNOS; 1:250, cat# 610333, BD Biosciences), COX1 (1:500; cat# 35-8100, Invitrogen), and COX2 (1:250; cat# 610204, BD Biosciences) were used. The antibody specificities for APP, BACE1, and eNOS were verified in knockout mice obtained from The Jackson Laboratory (stock #004133, stock #004714, and stock #002684, respectively; Supplementary Figure 5). In addition, C57BL/6J mouse heart was used as positive control for COX1, and whole cell lysate RAW 264.7 (cat# ab7187, Abcam) was used to identify COX2 and ADAM10 bands (Supplementary Figure 6). All blots were reprobed with β-actin antibody (1:50,000, A5316, Sigma). Densitometry analyses were performed in Odyssey Fc imaging system with Image Studio^™^ 5.2 software (Li-Cor Biotechnology, Lincoln, NE).

### Statistical analysis

All results are presented as mean ± standard error of mean (SEM; n indicates the number of mice used per experiment). The efficacy and potency of the drugs were determined using LabChart Pro Dose Response Module (ADInstruments Inc., Colorado Springs, CO). Concentration-response curves were compared by analysis of variance (ANOVA) for repeated measurements followed by Bonferroni's correction as described [29]. Two-way ANOVA followed by Tukey’s HSD test and unpaired t-test were used for multiple and single comparison, respectively (JMP Pro 14.1 software; SAS Institute, Cary, NC). Differences of P<0.05 were considered statistically significant.

## Supplementary Material

Supplementary Figures
