# Supplementary material for: Endothelium-specific deletion of amyloid-β precursor protein exacerbates endothelial dysfunction induced by aging
Source: Aging (Albany NY). 2021 Aug 12;13(15):19165–85. doi: 10.18632/aging.203401 (PMC8386539; doi:10.18632/aging.203401)
Supplement: Supplementary Figures [file aging-13-203401-s001.pdf]

SUPPLEMENTARY FIGURES

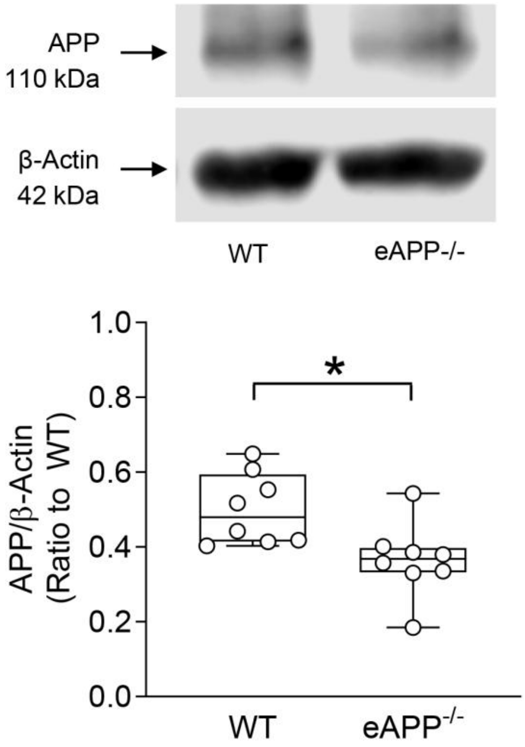

Supplementary Figure 1. APP expression in the aortas of young wild-type (WT) littermates and eAPP$^{-/-}$ mice. Western blots were performed in separate studies, and results are the relative densitometry compared with β-actin protein. All data are representing box plots with whiskers showing the median, 25th to 75th percentiles, and min-max range (n=8). *P<0.05 vs. WT littermates (unpaired t-test).

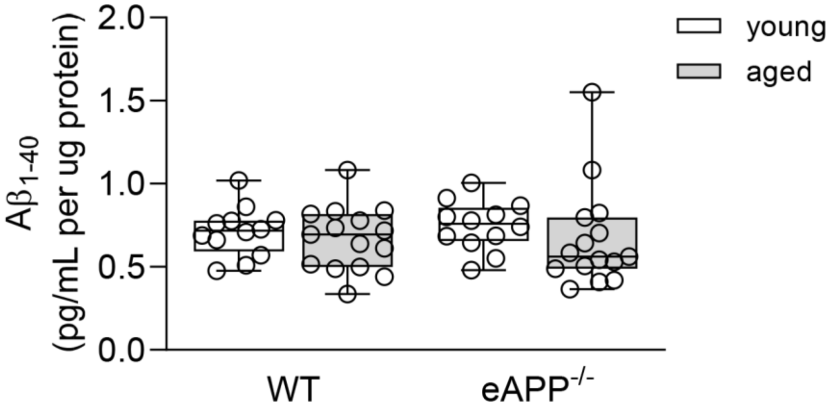

Supplementary Figure 2. Effects of aging on *ex-vivo* amyloid-β 1-40 (Aβ$_{1-40}$) secretion from wild-type (WT) littermates and eAPP$^{-/-}$ mice aortas. The supernatants were collected and analyzed for Aβ$_{1-40}$ levels. The results were normalized against tissue protein levels (n=12 per group for young WT littermates and eAPP$^{-/-}$ mice and n=15 per group for aged WT littermates and eAPP$^{-/-}$ mice. All data are representing box plots with whiskers showing the median, 25th to 75th percentiles, and min-max range. P>0.05 (two-way ANOVA followed by Tukey's HSD test).

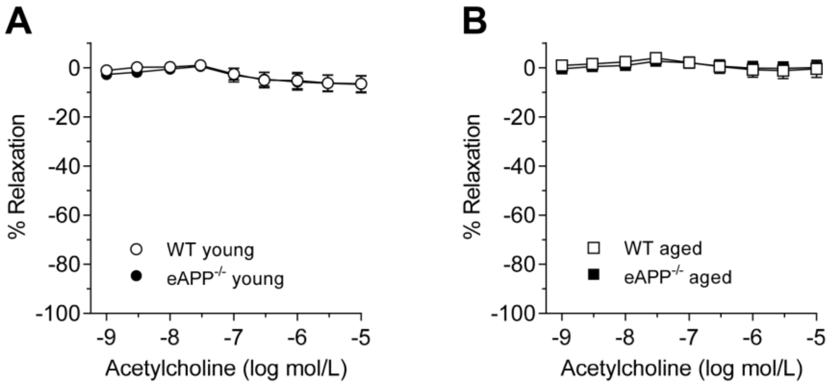

Supplementary Figure 3. Effects of L-NAME ($3x10^{-4}$ mol/L) on endothelium-dependent relaxations to acetylcholine in young (**A**; n=9 per group) and aged (**B**; n=7 per group) wild-type (WT) littermates and $eAPP^{-/-}$ mice aortas in the presence of indomethacin (10^{-5} mol/L). Results are shown as mean ± SEM and expressed as percent relaxation from submaximal contractions to $PGF_{2\alpha}$ ($3x10^{-6}$-$8x10^{-6}$ mol/L).

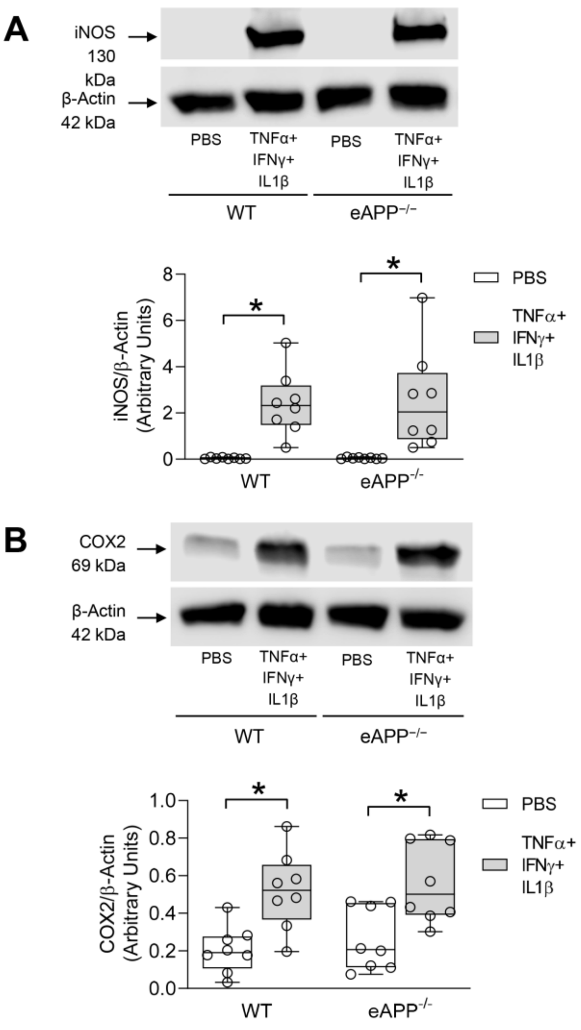

Supplementary Figure 4. Effects *ex-vivo* treatment for 24 hours with cytokine cocktail (consisting TNFα, IFNγ, and IL-1β) on iNOS (**A**, n=8 per group) and COX2 (**B**, n=8 per group) protein expressions of young wild-type (WT) littermates and $eAPP^{-/-}$ mice aortas. Western blot results are the relative densitometry compared with β-actin protein. All results are representing box plots with whiskers showing the median, 25th to 75th percentiles, and min-max range. * P<0.05 versus young mice of same strain (two-way ANOVA followed by Tukey's HSD test).

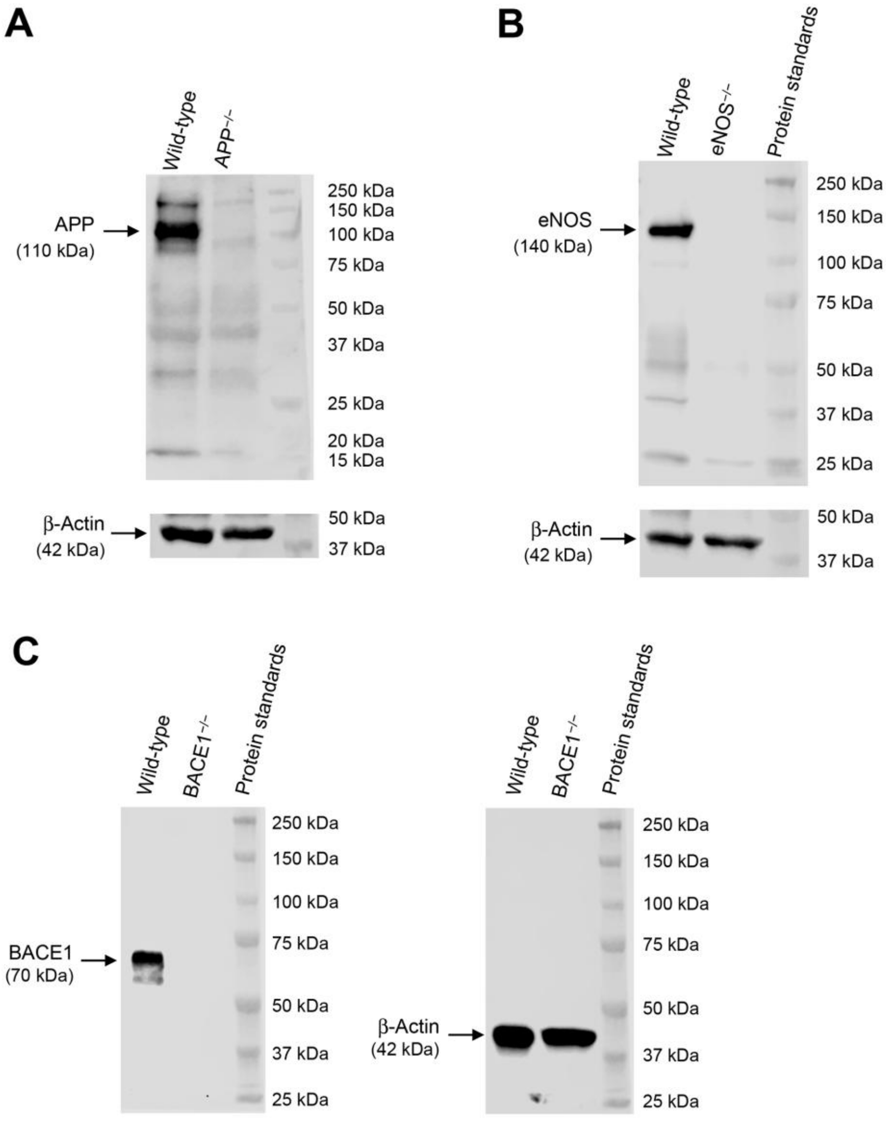

Supplementary Figure 5. Western blot analyses for validation of primary antibodies. (**A**) The selectivity of APP antibody was examined in APP$^{-/-}$ mice aortas (Stock no. 004133, The Jackson Laboratory). (**B**) The selectivity of eNOS antibody was examined in eNOS$^{-/-}$ mice aortas (Stock no. 002684, The Jackson Laboratory). (**C**) The selectivity of BACE1 antibody was examined in BACE1$^{-/-}$ mice aortas (Stock no. 004714, The Jackson Laboratory). As loading controls, all blots were reprobed with β-actin.

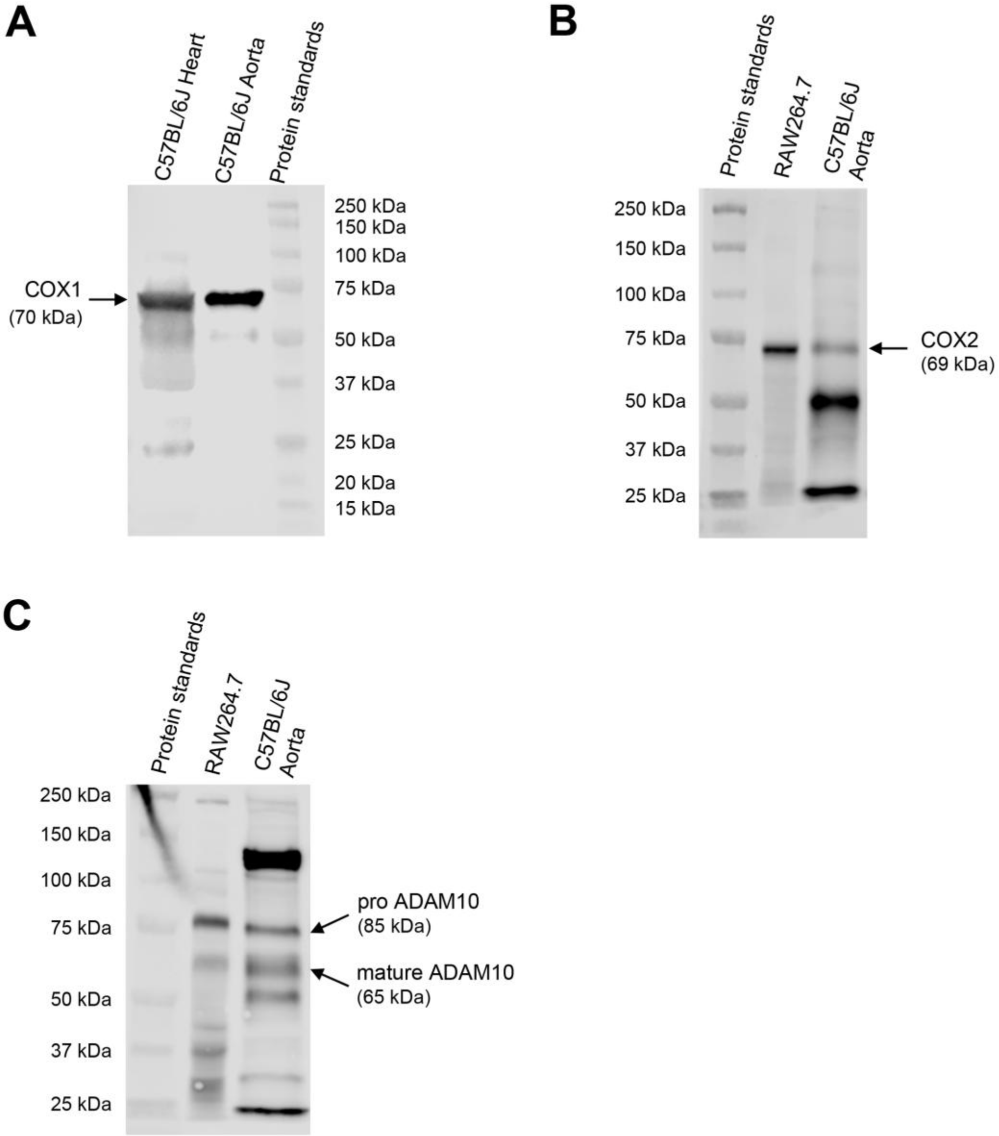

Supplementary Figure 6. Western blot analyses for validation of primary antibodies. (**A**) Wild-type mouse heart was used as positive control to identify COX1 band. (**B**) RAW264.7 whole cell lysate (no. ab7187, Abeam) was used as positive control for COX2 antibody. C57BL/6J aorta was incubated with cytokine cocktail (consisting TNFα, IFNγ, and IL-1β) for 24 hours (see method section for details) to induce COX2 expression. (**C**) Positive control RAW264.7 whole cell lysate (no. ab7187, Abeam) was used for selectivity of pro- and mature forms of ADAM10 bands.
